# Preparation and Rheological Behavior of Temperature-Resistant Hydroxypropyl Guar Gum Supramolecular Hydrogels Based on Dynamic Borate Ester Bonds

**DOI:** 10.3390/gels12090827

**Published:** 2026-09-10

**Authors:** Yikai Xing, Yongfei Li, Songwei Li, Bin Liu, Kai Gao, Chengjun Wang, Weiwei Han, Qian Wang, Yanling Wang

**Affiliations:** 1 College of Chemistry and Chemical Engineering, Xi’an Petroleum University, Xi’an 710065, China; 18291741772@163.com (Y.X.); 210104@xsyu.edu.cn (C.W.); hanweiwei@xsyu.edu.cn (W.H.); wangqian@xsyu.edu.cn (Q.W.); 2 Engineering Research Center of Oil and Gas Field Chemistry, Universities of Shaanxi Provence, Xi’an Petroleum University, Xi’an 710065, China; 3 The Eleventh Oil Production Factory, Petro China Changqing Oil Field Company, Qingyang 710021, China; lisongwei_cq@petrochina.com.cn; 4 CCDC Changqing Downhole Technology Operation Company, Xi’an 710021, China; cj_lbin@cnpc.com.cn (B.L.); cj_gaok@cnpc.com.cn (K.G.); 5 College of Petroleum Engineering, China University of Petroleum (East China), Qingdao 266580, China; wangyl@upc.edu.cn

**Keywords:** guar gum, water-based fracturing fluid, organic boron crosslinker

## Abstract

The increasing depth of oil wells and associated elevated formation temperatures pose significant challenges to conventional crosslinked polymer gels used in hydraulic fracturing, as viscosity degradation severely impairs proppant transport and stimulation efficiency. In this study, a thermally stable organic boron crosslinker was developed and evaluated in combination with hydroxypropyl guar gum (HPG). The rheological performance of the crosslinked gel system was systematically investigated under high-temperature shearing conditions. At a guar gum concentration of 0.5 wt% and a crosslinker-to-gum ratio of 100:0.5, the system maintains a viscosity of 500 mPa·s at 120 °C and 170 s^−1^, and the viscosity remains above 300 mPa·s after 60 min of continuous shearing, demonstrating outstanding thermal resistance. (For 0.3 wt% HPG, the optimal crosslinking ratio is 100:0.3; for 0.5 wt% HPG, the optimal ratio is 100:0.5). This gel formulation achieves efficient proppant transport with minimal additives. It maintains static proppant suspension for up to 48 h. After breaking, the fluid viscosity drops to approximately 5 mPa·s. With the addition of a flowback aid, surface tension can be reduced to as low as 16.29 mN/m, while the formation matrix permeability damage rate is limited to only 20.76%. Additionally, the gel features rapid breaking and low residue generation, making it highly suitable for high-temperature fracturing operations.

## 1. Introduction

With the continuous decline in global recoverable conventional oil and gas resources, the development of deep and unconventional reservoirs—such as tight oil, shale gas, and carbonate formations—has become a core strategic area for sustaining hydrocarbon reserves and production [1]. However, the geological characteristics of these deep formations pose severe engineering challenges. Specifically, increased burial depth raises the geothermal gradient, resulting in bottom-hole temperatures typically reaching 120 °C [2,3]. Such thermal environments impose rigorous demands on the thermal stability, shear resistance, and rheological retention of hydraulic fracturing fluid systems, properties that are critical for effective reservoir stimulation and proppant placement [1,4].

Among fracturing fluid systems, water-based fluids remain the preferred choice in industrial practice owing to their cost-effectiveness, environmental compatibility, and high pumping efficiency [5]. Hydrophilic polymers, particularly natural guar gum and its derivative hydroxypropyl guar gum (HPG), are widely employed as thickening agents due to their rapid solubility, strong thickening capability, and controllable break performance [1]. Nevertheless, HPG exhibits structural vulnerability under elevated thermal conditions. When exposed to 120 °C for extended periods, glycosidic bonds in the polysaccharide main chain undergo thermo-oxidative degradation and acid-/base-catalyzed hydrolysis [6,7]. This degradation results in a rapid decline in apparent viscosity, leading to premature fluid thinning, severe loss of proppant-carrying capacity, and potential operational failure [8].

To mitigate thermal degradation and maintain fluid integrity during pumping operations at 120 °C, chemical crosslinkers are indispensable. Crosslinkers react with hydroxyl groups on HPG polymer chains to construct dynamic three-dimensional networks, thereby enhancing apparent viscosity, thermal stability, and shear resistance [9,10]. Inorganic borate compounds (e.g., borax and boric acid) have long served as standard crosslinkers; however, they exhibit a distinct performance boundary at 120 °C. At this temperature, B–O coordination bonds undergo accelerated thermal dissociation due to intensified molecular motion [11,12]. This disruption leads to gel network collapse, rheological degradation, and syneresis, making 120 °C the upper reliability limit for traditional boron-crosslinked systems [12,13].

To achieve long-term rheological stability at 120 °C, advanced crosslinking technologies have been developed, including single and composite delayed crosslinkers incorporating organic boron, organic zirconium, or organic titanium compounds [14,15,16]. Introducing tailored organic ligands enables precise modulation of crosslinking kinetics to prevent premature surface crosslinking, while multi-metal coordination (such as boron–zirconium hybridization) reinforces network stability at 120 °C [17,18]. Furthermore, nanoscale crosslinkers—such as boron-functionalized colloidal silica and organic boron-functionalized nanocellulose—markedly enhance the thermal and shear resistance of HPG fracturing fluids under 120 °C conditions [19,20,21,22,23], successfully resolving the rheological stability bottleneck at this target temperature [24].

Developing a robust crosslinking system tailored for 120 °C reservoirs requires a multi-faceted balance among competing engineering parameters [25]. The system must maintain thermal stability and shear recovery under dynamic flow, provide optimal fluid-loss control, ensure thorough and residue-free gel breaking post-treatment, and minimize formation damage in sensitive reservoirs [8,26,27].

Based on these requirements, this study presents the synthesis and characterization of a novel temperature-resistant crosslinker specifically optimized for hydroxypropyl guar gum in 120 °C formations. By examining the high-temperature rheological behavior, dynamic bond equilibrium, and network integrity of the low-dosage crosslinked gel at 120 °C, this work aims to elucidate the underlying stabilization mechanism and establish the practical application value of the system for efficient, low-damage reservoir stimulation [28,29,30].

## 2. Results and Discussion

### 2.1. Preparation of Organoboron Crosslinker

#### 2.1.1. Selection of Organoboron Ligand Ratio

**Figure 1:**
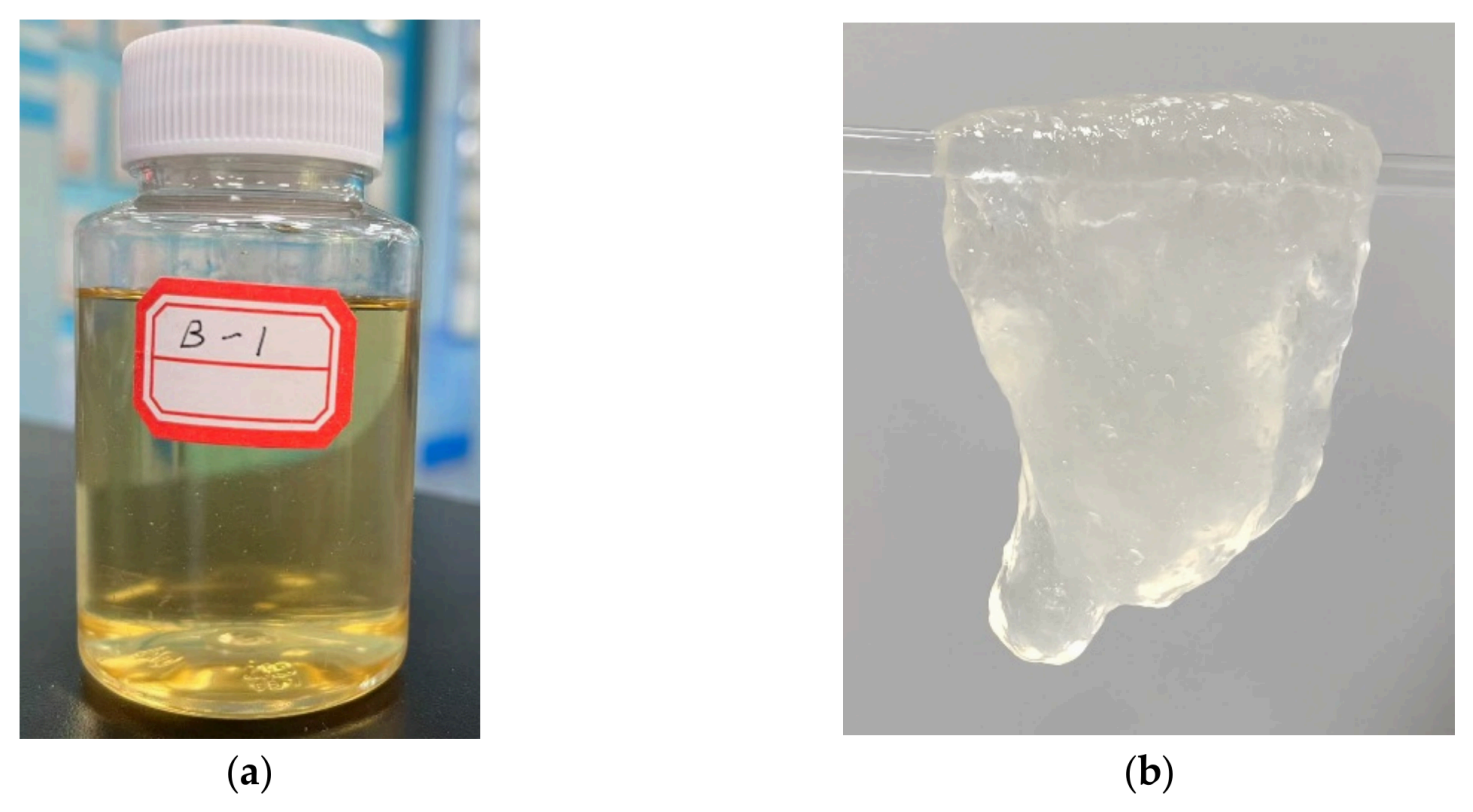
Macroscopic morphologies of the crosslinking system: (**a**) the prepared boron crosslinking agent (sample B-1), presenting as a clear and yellowish solution; (**b**) the crosslinked gel formed with the 0.3% HPG solution, demonstrating a robust three-dimensional network and excellent lip-hanging ability.

To construct HPG-based hydrogels with controllable rheological behavior, the precise regulation of the stoichiometric ratio of ligands in the crosslinker is critical, as it governs both the assembly pathway of the supramolecular network and the crosslinking kinetics. The ligands not only serve as buffers to modulate the local acidic/alkaline microenvironment of the crosslinking system, but also regulate the crosslinking reaction rate through steric hindrance effects. Based on the data presented in Table 1 and Table 2, this study systematically evaluated eleven organic boron crosslinking systems with different ligand ratios. Macroscopic sol–gel transition tests combined with rheological observations revealed that among the eleven formulations, the crosslinked products of formulations 5, 6, 7, 9, 10, and 11 exhibited pronounced fiber-forming ability (i.e., macroscopic “stringing” or “tack” characteristics). This phenomenon intuitively reflects that, after surpassing the gelation threshold, efficient crosslinking bridging occurs among the polymer long chains, successfully constructing a continuous and dense three-dimensional topological network. Consequently, the resulting hydrogels demonstrate excellent creep resistance and high viscoelasticity (Figure 1b).

From the perspective of supramolecular assembly at the microscopic level, the aforementioned favorable ligand microenvironment effectively promotes the condensation reaction between the active crosslinking ions and the cis vicinal dihydroxyl groups on the galactose residues of the HPG side chains, resulting in the formation of high density dynamic covalent crosslinking sites (boronate ester bonds). Based on comprehensive considerations of sol–gel transition kinetics and macroscopic gel strength, a pale yellow organic boron crosslinker with a pH of 7.5 was ultimately selected as the optimal candidate (Figure 1a).

**Figure 2:**
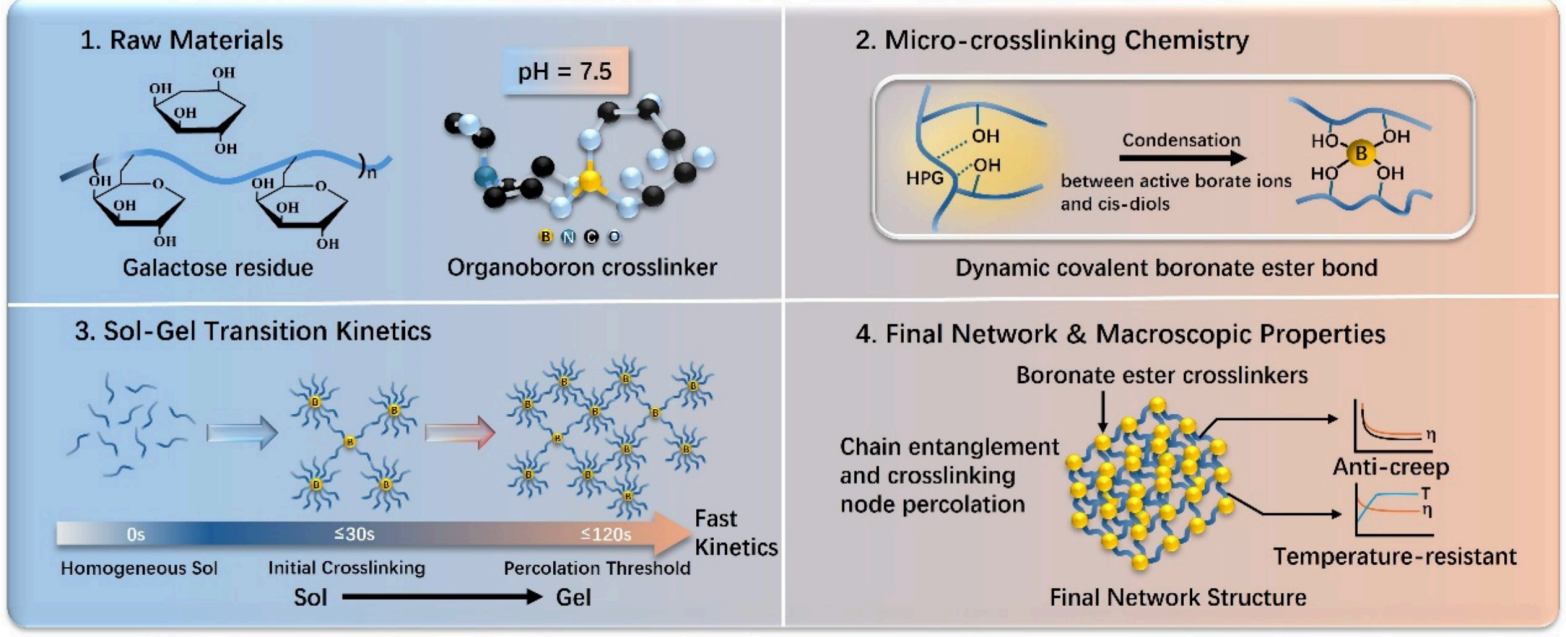
Schematic illustration of the sol–gel transition mechanism of the organoboron crosslinked HPG hydrogel. (**1**) Raw materials including HPG chains with cis-diol-containing galactose residues and organoboron crosslinkers at pH 7.5. (**2**) Microscopic crosslinking chemistry showing the condensation reaction between active borate ions and cis-diols to form dynamic covalent boronate ester bonds (B-O-C). (**3**) Macroscopic sol–gel transition kinetics, demonstrating the rapid transition from a homogeneous sol to initial crosslinking, and ultimately reaching the percolation threshold to form a gel within 120 s. (**4**) The final 3D topological network structure of the hydrogel, endowing the material with excellent creep resistance and high viscoelasticity.

The gelation mechanism is illustrated in Figure 2. The formation of the three-dimensional network structure of this gel system is not dominated by a single interaction, but is co-constructed by three distinct yet synergistic mechanisms:

(1) Dynamic Covalent Boronate Ester Bonds: This serves as the primary force for building the primary network skeleton of the gel. The cis-diols (or active hydroxyl groups) on the polymer chains undergo condensation reactions with borate ions from the crosslinker, forming stable B–O–C covalent crosslinking bonds. Owing to the dynamic reversibility of boronate esters, it endows the gel system with excellent shear-recovery and self-healing capabilities.
(2) Coordination Interactions: The stability of the crosslinking center is modulated through intramolecular coordination (e.g., the N→B coordination bond formed between the triethanolamine ligand and the central boron atom). Although this coordination does not directly connect two polymer chains, it effectively protects the central boron atom, controls the release rate of the crosslinker (delayed crosslinking), and significantly enhances the thermal stability of the crosslinking centers at elevated temperatures.
(3) Hydrogen Bonding: Beyond the dominant covalent network, extensive non-covalent hydrogen-bond interactions are formed among the abundant unreacted hydroxyl groups on the polymer backbone, as well as between hydroxyl groups and water molecules. These hydrogen bonds act as secondary physical crosslinking points, further increasing the crosslinking density of the system, dissipating external stress, and thereby substantially improving the overall viscoelasticity and mechanical strength of the gel.

When mixed with a 0.3 wt% HPG base fluid, this optimized system exhibits an exceptionally high kinetic response rate: the initial crosslinking time is effectively controlled within 30 s, and a complete sol–gel transition is achieved within 120 s. Such efficient gelation kinetics indicate that the crosslinker can rapidly induce chain entanglement and percolation of crosslinking points among polymer chains, facilitating a swift phase transition from a viscous fluid to an elastic hydrogel [31]. This ensures a high degree of structural integrity and stability of the gel network.

#### 2.1.2. IR and ^1^HNMR Spectral Results

**Figure 3:**
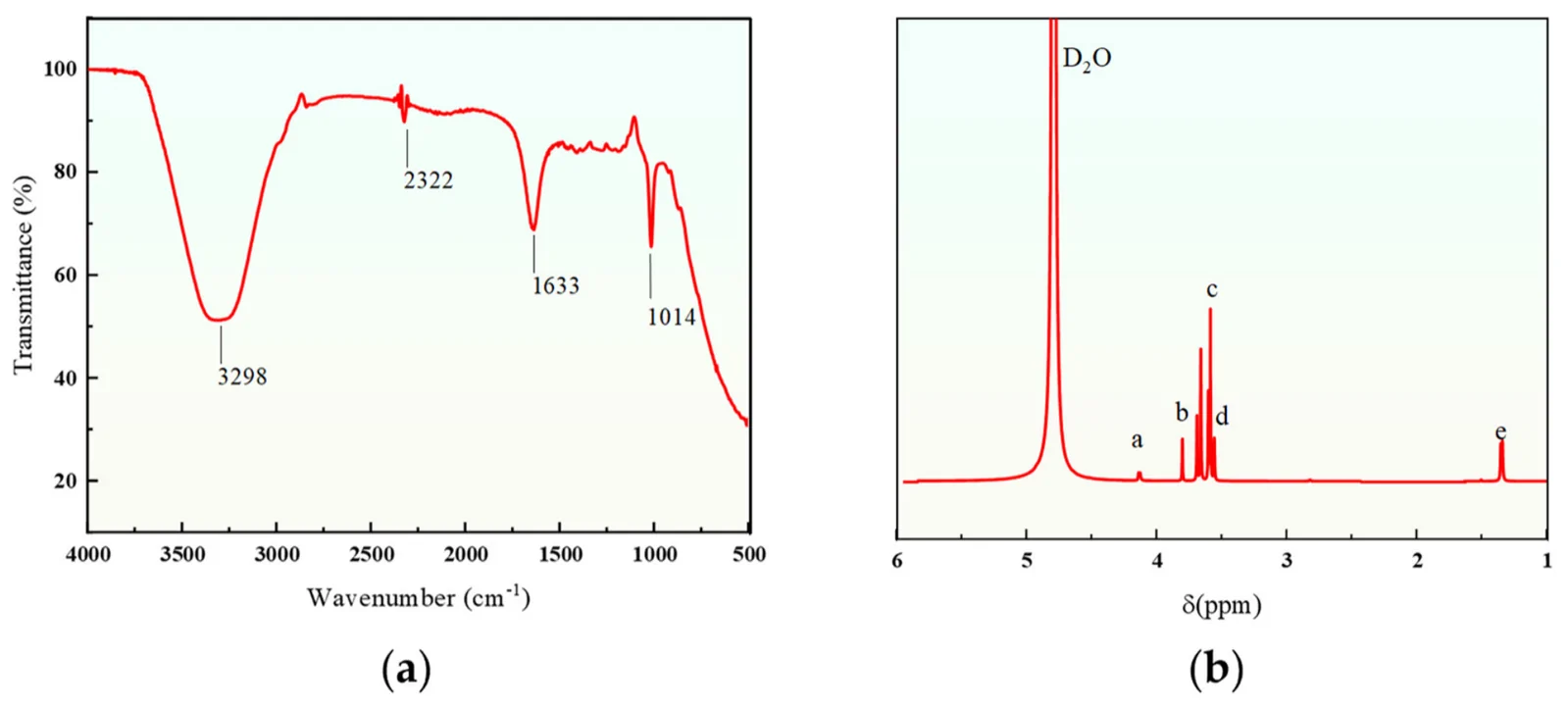
(**a**) ^1^H-NMR spectrum (400 MHz, D_2_O) and (**b**) FT-IR spectrum of the multi-ligand organoboron crosslinker synthesized via alkali-catalyzed condensation and coordination. In Figure 3b, peak **a** is assigned to the methine proton directly attached to the hydroxyl group in the auxiliary ligand; peak **b** is attributed to the proton generated by the acetal/hemiacetal reaction between glyoxal (as a small‑molecule bridging agent) and the polyols; peak **c** is assigned to the magnetically non‑equivalent methylene protons induced by the coordination of triethanolamine (TEOA) with the central boron atom; peak **d** is attributed to the vicinal diol protons on the sorbitol backbone in the crosslinked network, as well as to the uncoordinated TEOA protons present in the system; peak **e** is tentatively assigned to the methyl protons at the terminal end of the auxiliary ligand.

To deeply elucidate the microscopic chemical structure and coordination behavior of the novel multi-ligand organoboron crosslinker, this study systematically characterized it using proton nuclear magnetic resonance (^1^H-NMR) and Fourier-transform infrared (FT-IR) spectroscopies. As illustrated in Figure 3b, the ^1^H-NMR spectrum comprehensively reveals the chemical environment and complexation configuration of the multi-ligand crosslinker in the alkaline aqueous system:

Acetal network construction: The singlet (s, 1H) observed at δ = 3.80 ppm is attributed to the –CH(OR)_2_ protons generated by the acetal/hemiacetal reaction between glyoxal and polyols. This signal mutually corroborates with the absence of the free aldehyde C=O stretching vibration peak around 1720 cm^−1^ in the FT-IR spectrum (Figure 3a). This confirms that glyoxal, acting as a small-molecule bridging agent, has been completely converted, successfully pre-crosslinking the sorbitol molecules into a hydroxyl-rich network ligand with a larger spatial volume.

N→B coordination and bidentate/tridentate chelation: The characteristic doublet (d, J = 11.7 Hz, 8H) at δ = 3.67 ppm is assigned to the magnetically non-equivalent –CH_2_– protons induced by the N→B coordination between triethanolamine (TEOA) and the central boron atom. In the FT-IR spectrum, a strong absorption peak is observed at 1014 cm^−1^. While this peak alone is not sufficient to unequivocally establish a specific B–O–C environment due to potential overlap with typical C–O stretching vibrations, it remains consistent with the asymmetric stretching of B–O–C/C–O bonds. Consequently, when evaluated in conjunction with the ^1^H-NMR data, this FTIR feature strongly supports the premise that the central boron atom has transformed into a stable sp^3^-hybridized tetrahedral coordination anion.

Multi-crosslinked skeleton of polyhydroxyl ligands: The broad multiplet (m, 10 H) dispersed in the δ = 3.63–3.49 ppm region belongs to the vicinal diol protons of the sorbitol backbone alongside the uncoordinated portion of TEOA. The five-membered cyclic chelate ester formed by the vicinal diols of sorbitol and the boron atom significantly enhances the thermodynamic stability of the crosslinking coordination bonds.

In summary, a novel multi-ligand organoboron crosslinker with the potential for multiple network linkages was successfully constructed via alkali-catalyzed condensation and multiple coordination interactions. Comprehensive characterization by 1H NMR and FT-IR spectroscopies confirmed that the complex features an sp3-hybridized tetrahedral borate anion as the active core, for which triethanolamine constructs a crucial steric hindrance protective shell through strong N→B coordination. Simultaneously, glyoxal acts as a bridging agent to assemble the central-boron-chelated sorbitol ligands into a three-dimensional macromolecular network rich in free hydroxyl groups via exhaustive acetalization. This ingenious hybrid structural design, characterized by core–shell steric hindrance and multi-site acetal bridging, not only significantly enhances the thermodynamic stability of the crosslinking system but also provides abundant active sites for dynamic and reversible B-O bond exchange with polysaccharide gel systems (e.g., hydroxypropyl guar gum). Ultimately, this elucidates the molecular structural basis by which this crosslinker endows water-based fracturing fluid gels with excellent delayed crosslinking behavior and outstanding high-temperature shear resistance.

### 2.2. Influencing Factors of System Viscosity

#### 2.2.1. Effect of Standing Time on the Viscosity of HPG Base Fluid

**Figure 4:**
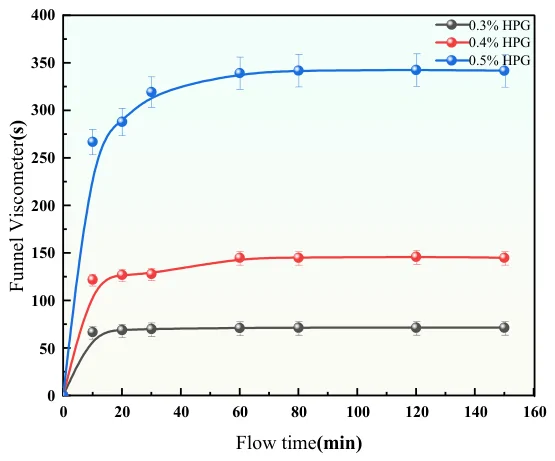
Swelling behavior of hydroxypropyl guar (HPG) base fluids at different concentrations (0.3 wt%, 0.4 wt%, and 0.5 wt%). The evolution of funnel viscosity is plotted as a function of time to indicate the hydration process.

Adequate swelling and hydration of polymer molecules are fundamental prerequisites for constructing a robust three-dimensional gel network. To evaluate this, the swelling behavior and hydration kinetics of HPG base fluids at various concentrations (0.3 wt%, 0.4 wt%, and 0.5 wt%) were continuously monitored, as depicted in Figure 4. During the initial swelling stage (the first 10–20 min), the funnel viscosity of all samples increased rapidly, demonstrating a strong hydrophilic hydration capacity. At this stage, water molecules rapidly penetrated the HPG polymer chains, driving chain unwinding and volume expansion. This rapid and thorough initial swelling effectively prevents the formation of unhydrated “fish-eye” agglomerates.

Subsequently, the hydration layer became fully established, and the rate of viscosity increase slowed down until reaching a swelling equilibrium, stabilizing at over 90% of the peak value. Furthermore, the final equilibrium viscosity and the time required to reach it were highly dependent on the polymer concentration. The 0.3 wt% HPG solution reached a stable funnel viscosity of approximately 71 s relatively quickly (within 30 min). In contrast, higher concentrations required longer times to achieve complete hydration, with the 0.5 wt% HPG solution taking up to 60 min to reach nearly 340 s. Considering the balance between a rapid hydration response and the baseline viscosity requirements, the 0.3 wt% HPG formulation exhibits optimal fast-swelling characteristics. Ultimately, this specific concentration provides a highly homogeneous conformational foundation for the subsequent efficient coordination crosslinking with the organic boron crosslinker.

#### 2.2.2. Effect of pH Value on the Viscosity of Hydroxypropyl Guar Gum

As shown in Table 3, pH exerts a decisive influence on the formation of the gel network structure. Under acidic conditions (pH = 4), the system failed to crosslink into a gel. In weakly acidic (pH 4–5) or strongly alkaline (pH 12–13) environments, the gels exhibited poor stringing performance, indicating a fragile network structure. At pH values ranging from 7 to 10 (neutral to weakly alkaline), the crosslinking time was moderate (18.71–37.31 s), and the gels displayed good stringing behavior. Although gels with acceptable stringing could be initially formed at pH 11–12, they underwent deterioration after standing for 30 min, which may be attributed to accelerated rearrangement of borate coordination bonds or degradation of the polysaccharide backbone at excessively high alkalinity. An appropriate pH favors rapid gel formation and enhances the overall strength of the system [32].

#### 2.2.3. Effect of Crosslinker Ratio on HPG Viscosity

Table 4, Table 5 and Table 6 systematically investigate the effects of crosslinker dosage on gelation characteristics at different HPG base fluid concentrations (0.3%, 0.4%, and 0.5%). At a low crosslinking ratio (100:0.2), regardless of the base fluid concentration, the gels exhibited relatively brittle physical properties and could not even be fully stringable, indicating that the crosslinking site density was insufficient to support a robust three-dimensional network. At high crosslinking ratios (100:0.5 to 100:0.6), the system underwent over-crosslinking, resulting in extremely short gelation times (e.g., only 10.27 s for the 0.5% HPG system) and consequent loss of stringability (brittle fracture). The optimal crosslinking ratio was found to be in the range of 100:0.3 to 100:0.4, within which all HPG concentrations exhibited favorable crosslinking kinetics and gel quality, representing the formulation window for achieving the best viscoelastic balance.

### 2.3. Rheological Behavior of the System

#### 2.3.1. Temperature Dependence of Viscosity for Different Systems

**Figure 5:**
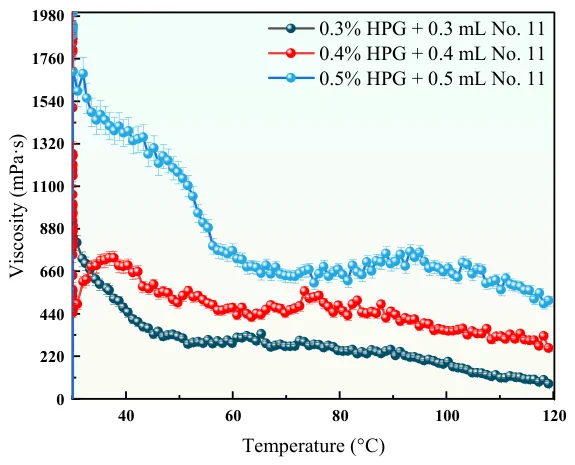
Viscosity–temperature curves of crosslinked HPG solutions at different concentrations.

Under high-temperature conditions in deep hydrocarbon reservoirs, intense molecular thermal motion typically triggers extensive disruption of non-covalent interactions or coordination bonds within supramolecular hydrogels, leading to macroscopic collapse of the three-dimensional topological network. Consequently, the thermodynamic stability of the crosslinked network is central to evaluating its engineering applicability. As can be visually observed from the viscosity–temperature curves in Figure 5, as the temperature gradually increases from ambient to 120 °C, the HPG crosslinked systems at different concentrations all undergo a certain degree of thermally induced thinning. However, no catastrophic viscosity drop or gel network disintegration occurs, indicating that the network integrity is largely preserved under these conditions.

Of particular note is the exceptional thermal resistance performance of the system with the highest polymer concentration (0.5% HPG + 0.5 mL of Boron crosslinker No. 11). During the initial heating stage, the apparent viscosity decreased somewhat, which is attributable to the rearrangement of polymer chain segments and enhanced solvation effects. However, after crossing the mid-temperature range around 60 °C, the viscosity decay trend slowed down significantly and the system exhibited remarkable robustness. When the temperature eventually reached and stabilized at the extreme thermal condition of 120 °C, the system still maintained a remarkably high retained viscosity of approximately 500 mPa·s.

From the perspective of microscopic thermodynamics and supramolecular chemistry, this outstanding resistance to thermal degradation profoundly reveals the fundamental restructuring of the gel network’s thermal stability imparted by the novel organic boron crosslinker. The coordination bonds formed between conventional inorganic borates and the cis-vicinal dihydroxyl groups of polysaccharide macromolecules are typical exothermic reversible reactions, and under high-temperature driving forces, the dissociation equilibrium shifts significantly toward the free state [33]. In contrast, the optimal organic boron crosslinker No. 11 developed in this study, through the introduction of specific ligand chemistry, exploits the electron-donating effects and pronounced steric hindrance of the organic ligands to firmly anchor the spatial conformation of the dynamic covalent boronate ester bonds. This microenvironmental optimization substantially elevates the thermal dissociation activation energy of the B–O crosslinking bonds. Therefore, even under high-energy thermal excitation at 120 °C, the crosslinking junctions can still maintain a remarkably high effective occupancy ratio, effectively suppressing polymer chain slippage and network disentanglement, thereby constructing a thermodynamic energy barrier against thermal depolymerization. This structural feature effectively overcomes the fatal drawback of conventional systems—namely, the facile dissociation of coordination bonds at 120 °C—and provides solid rheological support for the long-term stimulation of complex high-temperature reservoirs.

#### 2.3.2. Viscosity Retention Under Shear for Different Systems

**Figure 6:**
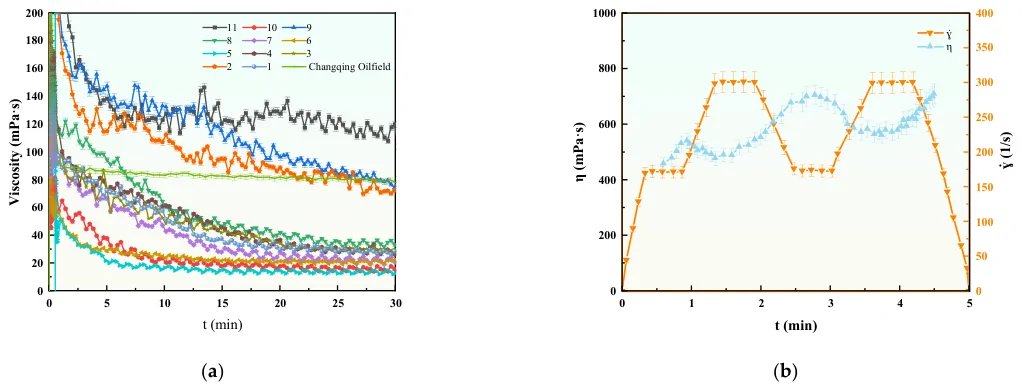
(**a**) Viscosity–time curves of 0.3% HPG solution crosslinked with different gelators under continuous shear (120 °C, 170 s^−1^). (**b**) Viscosity and shear rate as a function of time for the 0.3 wt% guar gum system crosslinked at a volume ratio of 100:0.3, subjected to cyclic shear at alternating shear rates of 170 s^−1^ and 300 s^−1^. The exceptional shear resistance is attributed to the strong dynamic reversible bonds (or specific interaction, e.g., hydrogen bonding, dynamic covalent bonds) between HPG and the crosslinker.

**Figure 7:**
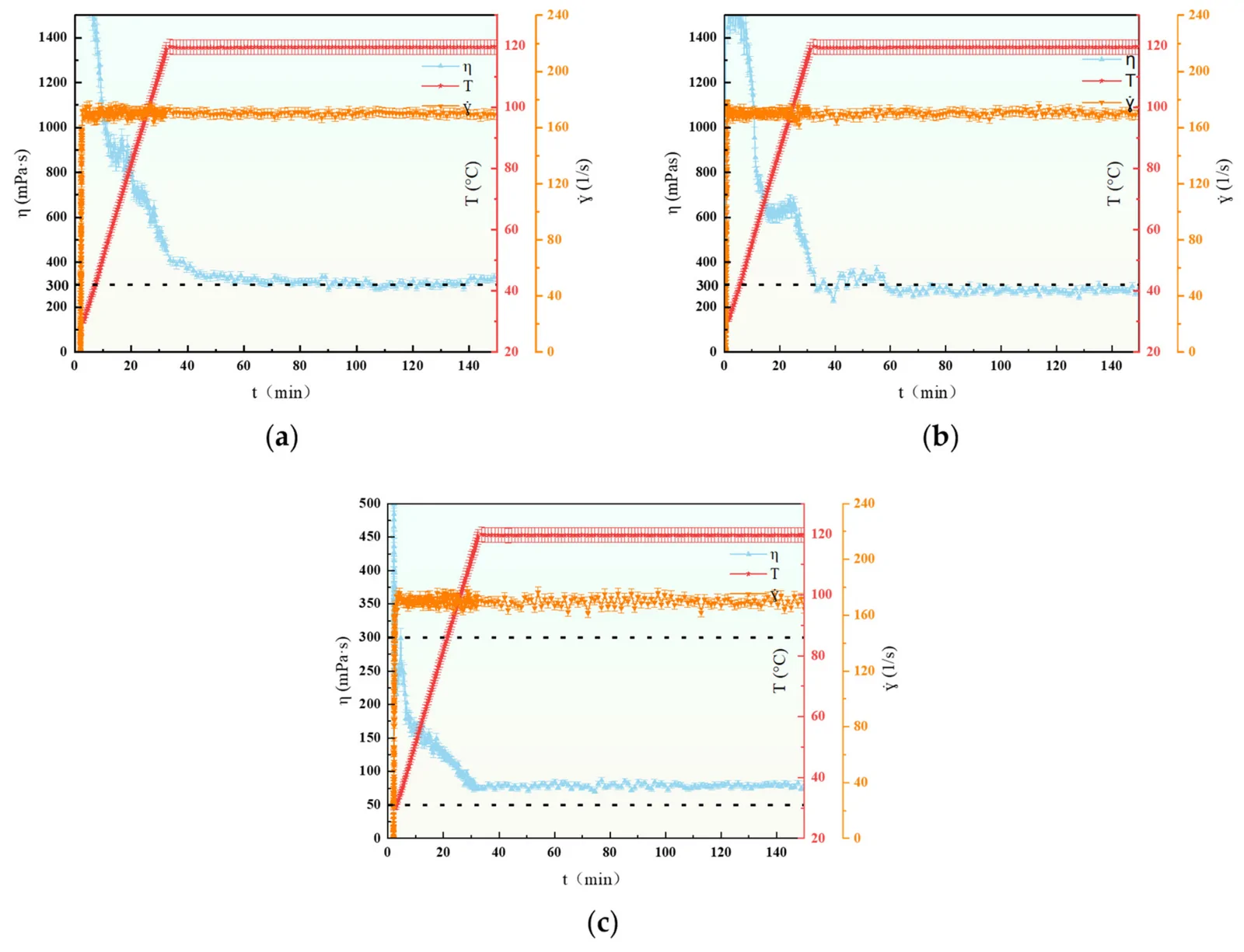
Dynamic rheological profiles of crosslinked HPG hydrogels under simulated high-temperature and constant-shear conditions (120 °C, 170 s^−1^). (**a**) 0.5 wt% guar gum + Crosslinker No. 11, crosslinking ratio 100:0.5; (**b**) 0.5 wt% guar gum + Crosslinker No. 11, crosslinking ratio 100:0.6; (**c**) 0.5 wt% guar gum + borate crosslinker used in a certain block of the Changqing Oilfield, crosslinking ratio 100:0.5. The blue, red, and orange lines represent the temporal evolution of viscosity (η), temperature (T), and shear rate ($\overset{\cdot }{\gamma }$), respectively. In Figure 7a,b, the black dashed lines represent a viscosity of 300 mPa·s; in Figure 7c, the black dashed lines represent viscosities of 50 mPa·s and 300 mPa·s.

The structural integrity and rheological response of hydrogels under extreme thermo-mechanical coupling fields are key determinants of their ability to maintain spatial topological networks and viscoelastic load-bearing capacity in deep formations. As evidenced by the static and dynamic rheological test data presented in Figure 6 and Figure 7, the crosslinked gel system exhibits outstanding thermal stability and mechanical shear tolerance. Taking 0.3 wt% guar gum as an example, the shear resistance of different crosslinkers was investigated at a crosslinking ratio of 100:0.3 (Figure 6a). The results show that the prepared No. 11 organoboron crosslinker exhibits a higher initial viscosity than the other samples and still maintains a viscosity of 120 mPa·s after 30 min of shearing. Meanwhile, the No. 11 crosslinker with 0.3 wt% guar gum at a crosslinking ratio of 100:0.3 was subjected to cyclic shear at alternating rates of 170 s^−1^ and 300 s^−1^. The results indicate that the gel system possesses a certain self-healing capability: the viscosity decreases under high shear, but rapidly recovers as the shear rate is reduced (Figure 6b). Subsequently, a system suitable for a formation temperature of 120 °C (0.5 wt% guar gum, crosslinking ratio 100:0.5) was evaluated. Under high-temperature conditions at 120 °C and continuous shearing at 170 s^−1^, the system displays an initial apparent viscosity of up to 500 mPa·s. Under the same conditions, a borate crosslinker used in a certain block of the Changqing Oilfield gives an initial viscosity of only 353.4 mPa·s, indicating that our system possesses a robust network crosslinking density and strong initial resistance to deformation (Figure 7).

To further evaluate the mechanical stability of the crosslinked networks under extreme wellbore conditions, a continuous thermal-shear test was conducted (Figure 7). The 0.5 wt% guar gum samples were subjected to a temperature ramp to 120 °C alongside a constant shear rate of 170 s^−1^. As depicted in Figure 7a, the hydrogel with a crosslinking ratio of 100:0.5 demonstrates excellent rheological stability. Despite the expected initial decrease in viscosity due to thermal thinning during the heating phase, the gel network reaches a robust equilibrium, maintaining a plateau viscosity of approximately 300 mPa·s for over 140 min.

When the crosslinking ratio is increased to 100:0.6 (Figure 7b), the equilibrium viscosity slightly decreases and exhibits more noticeable fluctuations. This phenomenon aligns with our previous observations regarding over-crosslinking. An excessive amount of crosslinker generates a highly densely crosslinked but brittle three-dimensional network. Under continuous, high-shear stress, this brittle network is more susceptible to localized structural rupture and permanent mechanical degradation, leading to a diminished macroscopic viscosity. Therefore, a strictly controlled crosslinking ratio (such as 100:0.5) is critical for balancing network density and elasticity to achieve optimal thermal and shear resistance. In comparison, the borate crosslinker used in the Changqing block (Figure 7c) maintains a plateau viscosity of only about 80 mPa·s over the same 140 min period. In contrast, Crosslinker No. 11 in this study exhibits superior shear resistance, with a viscosity retention rate of over 60%.

From the perspective of network dynamics and mechanical response (shear resistance), the system exhibited remarkable structural resilience throughout the 120 min period of intense shearing (170 s^−1^), with the final retained viscosity still stably maintained above 300 mPa·s (a viscosity retention rate of over 60%). This superior resistance to mechanical shear originates from the “fracture–recombination” equilibrium mechanism of the dynamic reversible covalent bonds within the gel network. Under a strong shear field, a portion of the stress-concentrated B–O crosslinking bonds undergo stress-induced reversible dissociation, effectively dissipating the mechanical energy imposed by external forces. Assisted by polymer chain diffusion and thermal motion, the dissociated borate ions rapidly recombine with the cis-vicinal dihydroxyl groups on adjacent polysaccharide chains, enabling in situ self-repair and topological network reorganization at the molecular level. This dynamic alternation of crosslinking junctions not only prevents irreversible mechanical scission of the polymer backbones but also endows the gel with an excellent energy dissipation mechanism and long-term rheological stability [34].

### 2.4. Evaluation of Engineering Applicability

#### 2.4.1. Proppant Suspension Ability

**Figure 8:**
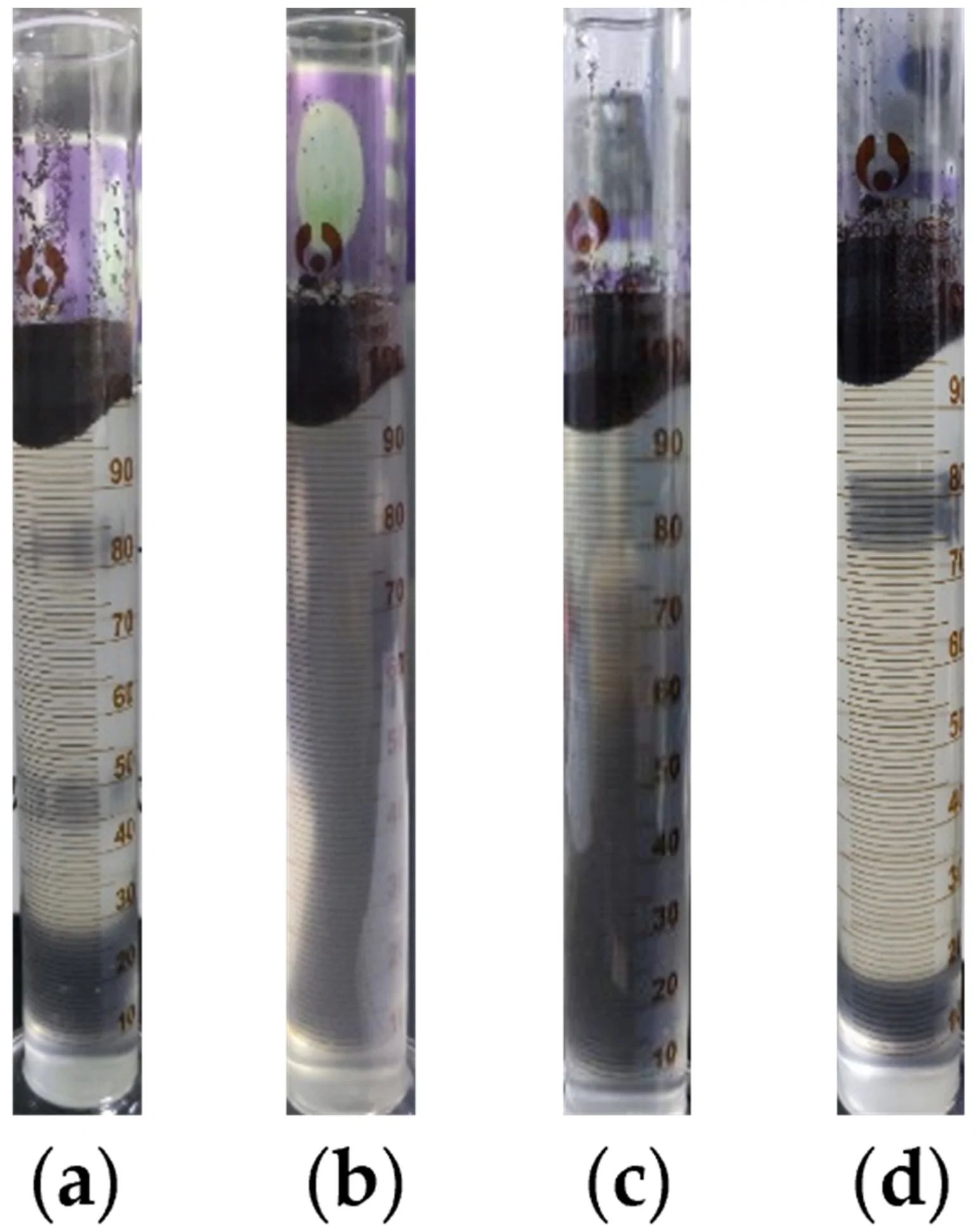
Visual observation of the static proppant suspension performance of the crosslinked hydrogel over time (sand volume fraction: 10 vol%): (**a**) 0 h; (**b**) 18 h; (**c**) 24 h; (**d**) 48 h.

**Figure 9:**
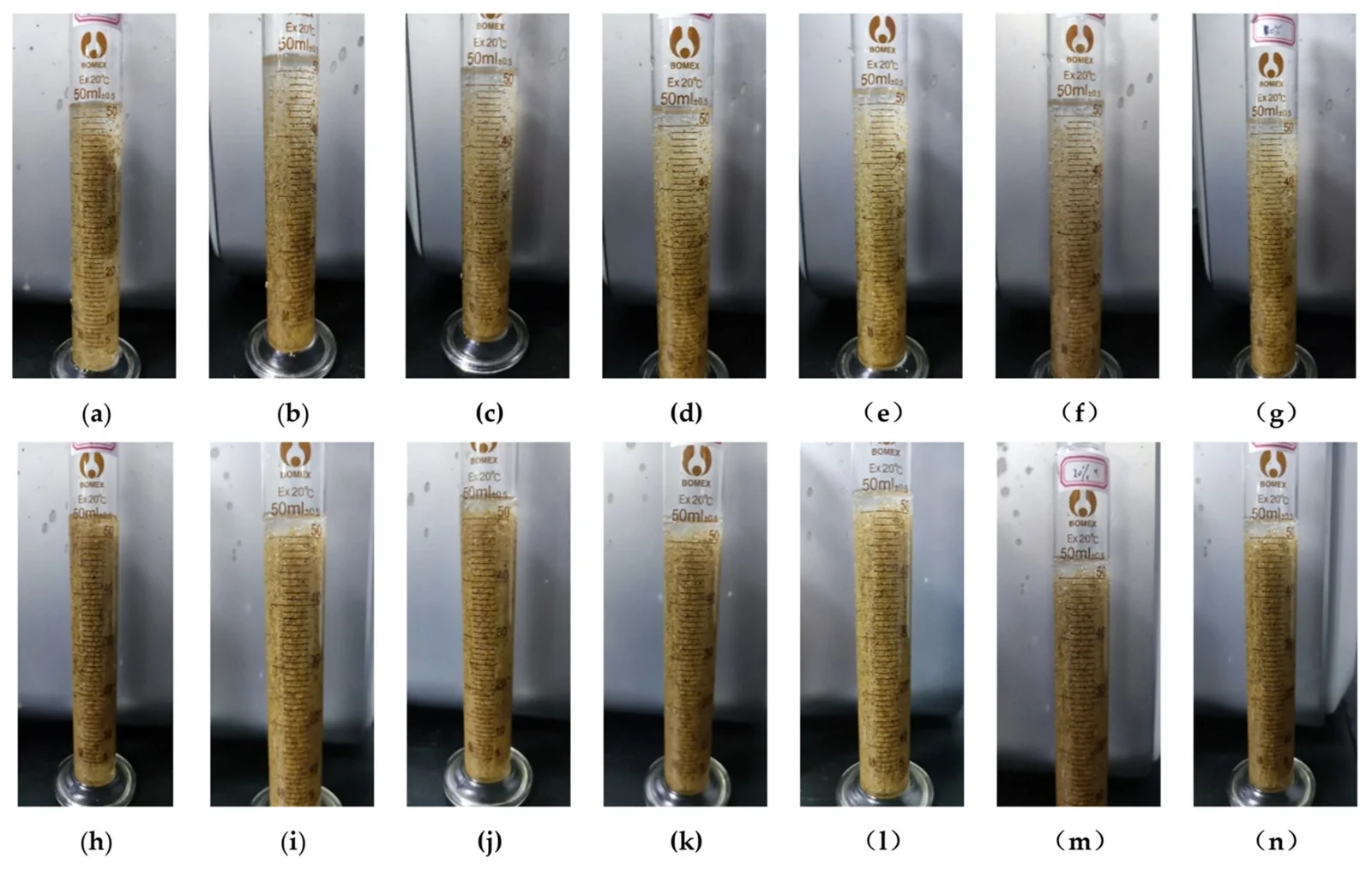
Visual observation of the static proppant suspension performance of the crosslinked hydrogel at different sand volume fractions over time: (**top**) 10 vol%; (**bottom**) 20 vol% ((**a**,**h**) 0 h; (**b**,**i**) 0.5 h; (**c**,**j**) 1 h; (**d**,**k**) 2 h; (**e**,**l**) 3 h; (**f**,**m**) 4 h; (**g**,**n**) 5 h).

Good and stable proppant suspension and transport capability are core indicators that determine whether a fracturing fluid can achieve effective proppant placement in complex fracture networks. Figure 8 and Figure 9 visually demonstrate the excellent proppant-suspending and transport behavior of this fracturing fluid system. In static proppant-settling tests at room temperature, the gel systems containing high proppant concentrations of 10 vol% and 20 vol% exhibited no significant solid particle settling or clear fluid separation after 48 h of quiescent storage. This result fully validates that the three-dimensional gel network formed within the system possesses a strong structural yield stress and outstanding viscoelasticity, enabling effective entrapment and encapsulation of high-density solid particles.

From an engineering application perspective, this static proppant suspension performance holds significant practical value for field operations. First, it satisfies the demanding requirements of high proppant concentration and high-rate pumping treatments. The system’s excellent suspension capability at a proppant loading of 20 vol% implies that higher concentrations of proppant can be safely pumped during field operations, thereby forming wider and higher-conductivity flow channels after fracture closure, which enhances single-well productivity. Second, it reduces operational risks on site. In actual fracturing processes, unexpected pump shutdowns often occur due to equipment failures or process requirements. The 48 h static proppant suspension stability of this system at room temperature ensures that, even during prolonged pump interruptions, the proppant remains stably suspended within the wellbore and fractures, effectively preventing catastrophic downhole events such as sand plugging or premature screen-out caused by rapid particle settling, thus broadening the safety window of the operation. Third, it enables deep proppant placement at distal fracture tips, effectively expanding the stimulated reservoir volume (SRV). The transport performance ensures that proppant does not prematurely settle as flow velocity decreases during migration along the main fracture into deeper formation zones. This allows the proppant to be smoothly conveyed into micro-fractures and far-end fracture regions, avoiding near-wellbore stacking, and thereby maximizing effective proppant placement at the distal ends, which lays a physical foundation for the long-term development of tight or ultra-low permeability reservoirs.

#### 2.4.2. Evaluation of Breaker Efficiency

The network disruption and degradation behavior of hydrogels after fulfilling their stress transfer tasks is key to evaluating whether efficient flowback can be achieved in tight porous media. As demonstrated by the degradation kinetics results in Table 7, this supramolecular gel system exhibits a highly sensitive oxidative degradation response to ammonium persulfate (APS). Upon thermal activation at 90 °C, APS undergoes homolysis to release highly reactive sulfate radical anions (SO_4_^−^·). These radicals precisely and efficiently attack the glycosidic bonds in the polysaccharide backbone, triggering random scission of the polymer main chains and complete disintegration of the three-dimensional topological network. With the introduction of only trace amounts of APS (0.001–0.08 wt%), the system undergoes a thorough gel–sol transition within 2 h. During this phase transformation, the macroscopic rheological characteristics of the system are fundamentally reversed, with an order-of-magnitude reduction in apparent viscosity, ultimately dropping to approximately 5 mPa·s, corresponding to a complete conversion into a very low-viscosity solution, and is significantly lower than the viscosity of the uncrosslinked 0.3 wt% guar gum base fluid (20 mPa·s). From the perspective of porous media flow mechanics, this efficient degradation of the crosslinked system and the extreme reduction in residual fluid viscosity endow the degradation products with superior microscopic mobility. The extremely low liquid viscosity not only eliminates the risk of retention and mechanical bridging of undegraded macromolecules in narrow pore throats, but also substantially reduces the shear friction along the flow path during fluid transport through the porous matrix. This thorough structural deconstruction ensures that the broken gel fluid can be rapidly and completely flowed back to the surface under a very low pressure gradient after fracturing operations, thereby providing a reliable rheological guarantee for maximizing the restoration of reservoir matrix conductivity [35].

#### 2.4.3. Surface Tension of the Broken Gel

After the gel network has undergone complete three-dimensional degradation, the phase-transport capability of the broken gel fluid in microporous media—namely, the flowback efficiency—is a critical indicator for evaluating the engineering applicability of this hydrogel system. As shown by the interfacial tension test results in Table 8, the gas–liquid surface tension of the neat broken gel fluid is 33 mN/m, which is significantly lower than that of ultrapure water (72.967 mN/m). This reduction indicates that the polymer chain fragments or amphiphilic small molecules generated from gel degradation undergo some degree of interfacial adsorption and self-assembly. Of particular note, upon the introduction of a trace amount (0.5 mL) of a flowback aid, the interfacial free energy is further disrupted, causing a sharp reduction in surface tension to an extremely low value of 16 mN/m.

From the perspective of interfacial hydrodynamics and based on the Young–Laplace equation, the capillary resistance encountered by fluids during transport in micro-/nanoscale pore-throat networks is positively and directly correlated with the liquid surface tension. The ultra-low surface tension of 16 mN/m can substantially weaken the capillary end-effects and significantly alter the wetting dynamics of the gas–liquid–solid three-phase contact line. This superior interfacial thermodynamic advantage endows the residual fluid with exceptionally high mobility, enabling it to readily overcome the seepage resistance within tight porous matrices. Therefore, this not only effectively alleviates the “water blockage” effect in deep reservoirs, but also ensures that the broken gel fluid can be rapidly and thoroughly flowed back from the fracture network to the surface, thereby minimizing secondary damage to the matrix pores.

#### 2.4.4. Core Permeability Damage Rate

**Figure 10:**
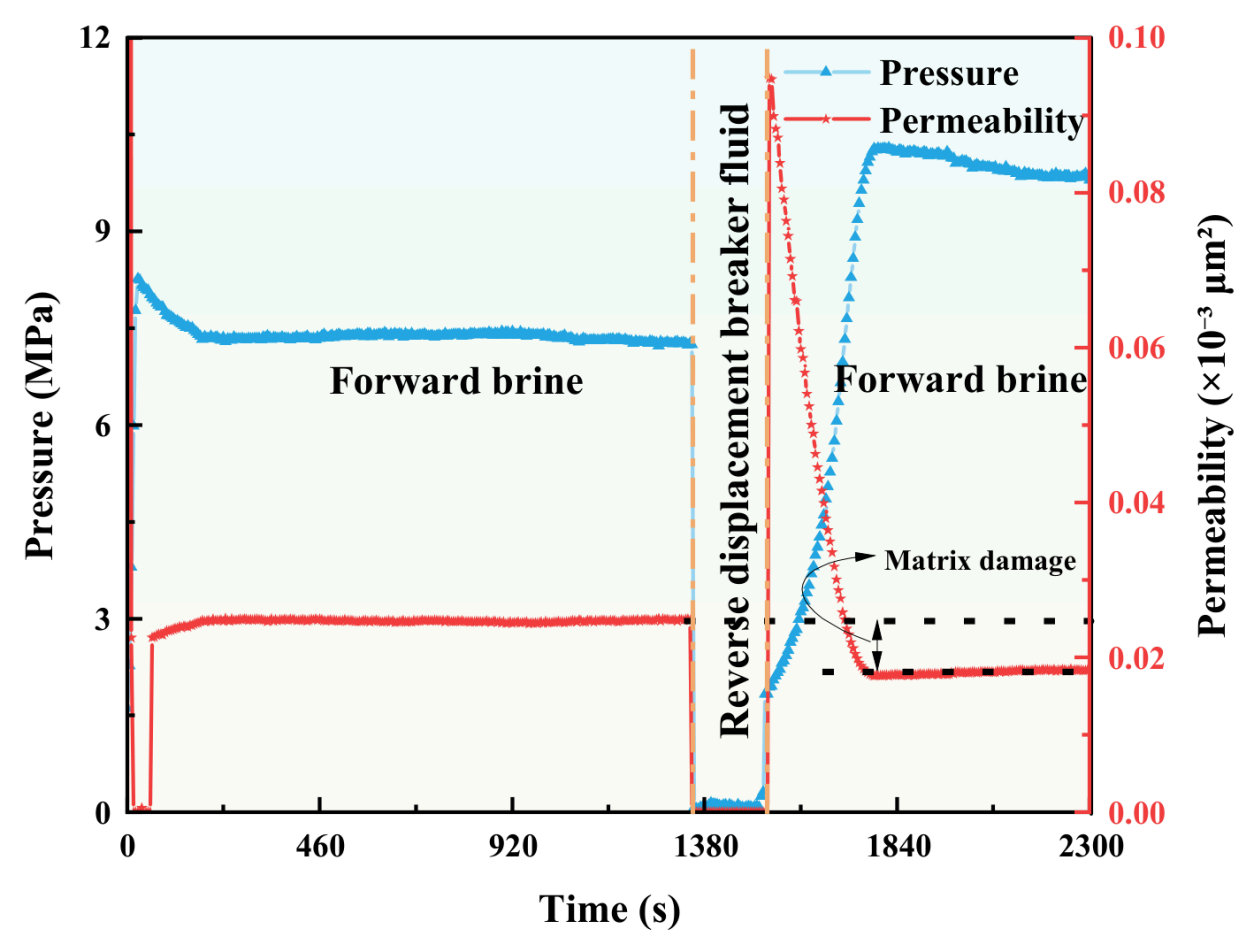
Dynamic core damage process showing permeability evolution as a function of time and applied pressure. The test was conducted at 90 °C using standard brine (2.0 wt% KCl + 5.5 wt% NaCl + 0.45 wt% MgCl_2_ + 0.55 wt% CaCl_2_) as the flowing medium. Parameters plotted include pressure (MPa), permeability (×10^−3^ μm^2^), forward brine injection pressure (MPa), and matrix damage rate (%). The damage rate remained nearly constant at approximately 20% during the later stage, indicating stable formation damage under the test conditions.

After gel breaking, the system leaves behind a certain amount of residue, and the broken gel fluid itself may cause formation damage and permeability reduction due to incompatibility with formation fluids. The core damage test results are presented in Table 9 and Figure 10. For the ultra-low permeability reservoir of the M2-2 interval (initial porosity of 8.534%, liquid permeability of 0.09125 × 10^−3^ μm^2^), static damage and dynamic displacement experiments with the broken gel fluid were conducted at 90 °C. Affected by filtrate invasion, the core permeability decreased to 0.07231 × 10^−3^ μm^2^ after damage, corresponding to a matrix permeability damage ratio of 20.76%. According to conventional reservoir sensitivity evaluation criteria, a damage ratio of approximately 20% falls within the weak to moderately weak damage category [35]. Considering that ultra-low permeability cores are highly sensitive to solid particles and liquid retention, the overall damage ratio of about 20% is at the boundary of weak damage. This indicates that the fracturing fluid system exhibits good gel-breaking performance, with low residue and minimal retention of macromolecular polymers, and shows good compatibility with formation fluids. Thus, while effectively creating fractures, the system can maximize the protection of reservoir matrix conductivity.

#### 2.4.5. Residue Concentration of the Broken Gel

In the engineering application lifecycle of hydrogel-based fracturing fluids, the thoroughness of degradation of the supramolecular crosslinked network after it has fulfilled its tasks of stress transfer and proppant transport directly determines the ultimate stimulation effectiveness of the reservoir. Combined with the degradation product analysis presented in Table 10, the degradation kinetics of this hydrogel system under thermal activation at 90 °C exhibit remarkably high conversion efficiency. The water-insoluble macromolecular residues (i.e., solid residue content) in the broken gel fluid are tightly controlled at an extremely low level of only 278–290 mg/L. From the perspective of polymer physics and chemistry, this indicates that both the boronate ester crosslinking junctions and the polysaccharide backbones within the network undergo highly complete scission during the oxidative degradation process, effectively suppressing the formation of hydrophobic macromolecular aggregates that would otherwise arise from incomplete network depolymerization.

In actual fracturing operations in tight/ultra-low permeability reservoirs, this thorough micro-scale structural deconstruction demonstrates highly promising macro-scale application value. The extremely low residue content effectively eliminates the risk of mechanical bridging by solid polymer debris in micro- and nano-scale pore throat networks and effectively prevents the irreversible deposition of compact degradation filter cake on fracture faces and rock matrix surfaces. This not only ensures that the system can maximize the preservation of the topological connectivity of the porous medium and the inherent permeability of the matrix, but also guarantees that the propped fractures maintain excellent long-term conductivity after closure. This rheological response, which combines the dual advantages of high-strength viscoelastic support and efficient degradation, meets the stringent engineering requirements for environmentally friendly hydrogel systems with ultra-low formation damage in modern oil and gas extraction, providing a reliable materials-based solution for efficient and long-lasting reservoir stimulation under complex geological conditions.

#### 2.4.6. Compatibility of the Breaker Fluid

**Figure 11:**
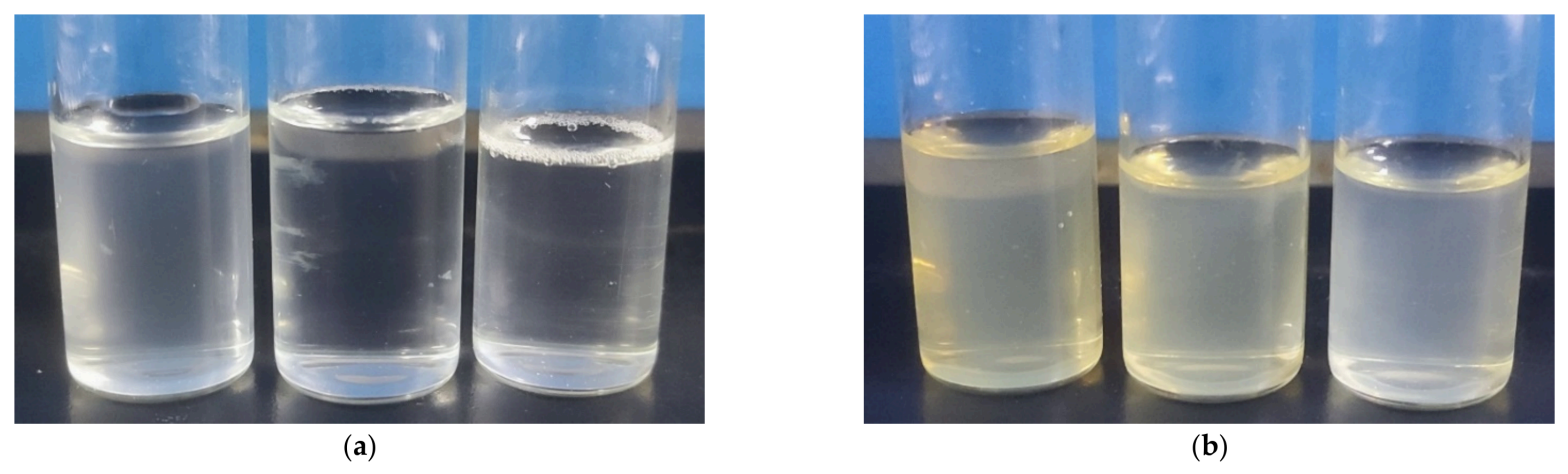
Compatibility of breaker fluid with formation water: (**a**) Qinghai formation water; (**b**) Sulige formation water. In each subfigure, the ratios of breaker fluid to formation water are 1:2, 1:1, and 2:1 from left to right.

The phase behavior and chemical compatibility between the degraded residual fluid—after complete deconstruction of the crosslinked hydrogel network—and the in situ reservoir fluids, particularly high-salinity formation water, are critical physicochemical indicators that determine whether the system can achieve “low permeability damage” flowback under realistic reservoir conditions. As shown by the compatibility test results in Figure 11, under simulated high-temperature formation conditions, the broken gel fluid, when mixed with high-salinity formation water at various volume ratios, consistently maintained a highly transparent and clear isotropic homogeneous state, with no occurrence of sol–gel phase separation, colloidal aggregation, solution turbidity, or secondary crystallization/precipitation.

Conventional inorganic boron or low-stability organic boron crosslinking systems, when mixed with high-salinity formation water rich in divalent metal cations (e.g., Ca^2+^, Mg^2+^), are prone to non-specific complexation between free borate ions and multivalent cations, resulting in the formation of insoluble inorganic borate scales. Meanwhile, the salting-out effect induced by high ionic strength may also cause hydrophobic aggregation and precipitation of incompletely degraded polymer segments. In this study, the excellent compatibility exhibited by the novel organic boron crosslinker and its degradation products is attributable to the strong complexation shielding effect of the organic ligands around the boron center, which effectively prevents non-specific binding between free boron ions and multivalent cations in formation water. Moreover, the small polysaccharide fragments generated from complete main-chain degradation maintain a high solvation capacity even under high ionic strength conditions, thereby ensuring the phase stability of the mixed system from a thermodynamic standpoint.

This high degree of chemical compatibility at both the microscopic and mesoscopic scales endows the hydrogel system with significant practical engineering value for the stimulation of deep/ultra-deep, high-temperature, high-salinity reservoirs. It effectively avoids the risk of inducing secondary inorganic scale or organic precipitates within micro-/nano-scale pore throats upon contact between the broken gel fluid and formation fluids, thereby eliminating the root causes of pore blockage and an aggravated Jamin effect. This not only safeguards the natural permeability pathways of the matrix and the high conductivity of propped fractures, ensuring efficient and thorough fluid flowback after fracturing, but also provides reliable material support for long-term reservoir protection in stimulation operations under complex and harsh formation conditions.

## 3. Conclusions

In this study, a novel organic boron crosslinker (optimal formulation No. 11) was successfully developed, using boric acid as the boron source and sorbitol and glyoxal as specific ligands. This crosslinker was combined with 0.3, 0.4, and 0.5 wt% hydroxypropyl guar gum (HPG) to construct supramolecular hydrogel systems with excellent rheological behavior (for 0.3 wt% HPG, the optimal crosslinking ratio is 100:0.3; for 0.4 wt% HPG, the optimal ratio is 100:0.4; for 0.5 wt% HPG, the optimal ratio is 100:0.5). Microstructural and gelation kinetic studies revealed that the ligand microenvironment effectively promoted the formation of high-density dynamic covalent crosslinking sites (boronate ester bonds). The system exhibited an exceptionally high response rate at pH 7.5, achieving a complete sol–gel transition within a crosslinking time of less than 30 s.

The hydrogel system demonstrated outstanding thermodynamic stability and mechanical shear tolerance. Under harsh shear conditions of 120 °C and 170 s^−1^ for 120 min, the retained viscosity remained stably above 300 mPa·s, which is primarily attributed to the efficient “fracture–recombination” self-healing mechanism of the dynamic reversible covalent bonds within the gel network. Engineering application evaluations confirmed that the gel system exhibits a static proppant suspension capability of up to 48 h at a high proppant loading of 20 vol%. Upon thermal activation at 90 °C, the introduction of trace amounts of ammonium persulfate (APS) enabled efficient and thorough network degradation, with the broken gel fluid viscosity dropping to approximately 5 mPa·s. With the assistance of a flowback aid, the surface tension was reduced to as low as 16.29 mN/m, and the solid residue content remained extremely low (<300 mg/L). Furthermore, the system exhibited excellent compatibility with high-salinity formation water. Core damage evaluation results showed a matrix permeability damage ratio of only 20.76%, falling within the weak damage category, thereby maximizing the protection of reservoir conductivity. This study provides a solid material foundation for long-term, low-permeability-damage stimulation of complex high-temperature, high-salinity deep hydrocarbon reservoirs.

## 4. Materials and Methods

### 4.1. Materials

Hydroxypropyl guar (HPG) powder was provided by Shandong Yousuo Chemical Technology Co., Ltd. (Linyi, China) Boric acid, sorbitol, glyoxal, triethanolamine, sodium hydroxide, and ammonium persulfate were all of analytical grade and purchased from Tianjin Kemiou Chemical Reagent Co., Ltd. (Tianjin, China). Deionized water was used in all experiments.

### 4.2. Experimental Methods

#### 4.2.1. Preparation of the Novel Organoboron Crosslinker and Gel System

The synthesis process of the novel organoboron crosslinker (No. 11) was as follows: sorbitol and boric acid were accurately weighed according to a specific stoichiometric ratio and dissolved in an appropriate amount of deionized water. Sodium hydroxide (NaOH) flakes were slowly added in batches to adjust the acid-base microenvironment of the system. After the solution cooled, a specific proportion of glyoxal was added, and continuous mechanical stirring was applied until complete dissolution. Finally, triethanolamine was added dropwise into the system for endpoint adjustment until the solution appeared pale yellow and the final pH stabilized at 7.5, yielding the target organoboron crosslinker.

The preparation method of the gel system, with reference to the standard (SY/T 7627-2021 Technical requirements of water-based fracturing fluid) [36], is as follows: hydroxypropyl guar gum (HPG) dry powder was slowly dispersed into deionized water and continuously hydrated for 60 min under constant-speed mechanical stirring to obtain an HPG base fluid with a mass fraction of 0.3 wt%. Subsequently, 100 mL of this base fluid was measured, and 0.3 mL of the aforementioned organoboron crosslinker was added under constant shear (the crosslinking volume ratio was set to 100:0.3). The crosslinking time of the system and the hanging morphology after the sol–gel phase transition were accurately recorded. Definitions: Crosslinking time refers to the time required for the vortex at the surface of the base fluid to disappear and for the liquid surface to rise slightly (i.e., to exhibit a slight protrusion). Gel time (i.e., the time needed to form a gel that can be lifted or hung) refers to the time required for the system to develop a gel state that can be picked up with a spatula or rod (i.e., a state capable of hanging morphology).

#### 4.2.2. Microstructural Characterization

The functional group structure of the crosslinker was characterized using a Fourier transform infrared spectrometer (FTIR, Nicolet 5700, Thermo Fisher Scientific, Waltham, MA, USA). The samples were prepared using the smear method, and the tested wavenumber range was 4000–400 cm^−1^. In addition, its backbone structure was analyzed using a nuclear magnetic resonance spectrometer (1H NMR, AVANCE NEO 400 MHz, Bruker, Billerica, MA, USA), with deuterium oxide (D_2_O) used as the dispersion solvent during the test.

#### 4.2.3. Rheological Behavior Evaluation

The rheological and shear resistance properties of the gel system were evaluated using an advanced rheometer system. In the viscosity–temperature test, the crosslinked gel was placed in the test fixture, and the system temperature was heated from room temperature to 120 °C at a constant heating rate. The variation in apparent viscosity with temperature was continuously recorded. The thermal shear resistance test was conducted at 120 °C with a constant shear rate of 170 s^−1^. The sample was subjected to a continuous dynamic shear test for 120 min. The viscosity changes before and after shearing were recorded, and the viscosity retention rate was calculated to evaluate the shear resistance and self-healing ability of the dynamic covalent bonds within the system.

#### 4.2.4. Engineering Application Performance Evaluation

Proppant suspension performance and gel breaking properties: To investigate the static proppant suspension performance of the system, proppants with volume fractions of 10 vol% and 20 vol% were uniformly mixed into the crosslinked gel (in accordance with the standard SY/T 5185-2024 Measure methods of water-based carrying fluid for gravel packing sand control) [37]. In the gel breaking experiment, ammonium persulfate (APS) with a mass fraction ranging from 0.001 wt% to 0.08 wt% was introduced into 100 mL of the crosslinked gel. The mixture was placed under a constant temperature of 90 °C to initiate oxidative degradation. The gel breaking time and the final viscosity of the residual fluid were recorded. After gel breaking, the interfacial tension of the original broken gel fluid and the residual fluid supplemented with 0.5 mL of flowback aid was measured. The broken gel fluid was vacuum-filtered, dried, and weighed to determine the insoluble residue content. Simultaneously, the broken gel fluid was mixed with high-salinity formation water and allowed to stand to observe its phase compatibility (in accordance with the standard SY/T 7627-2021).

Dynamic Core Damage Evaluation: A fully automatic core flooding system was used to evaluate the degree of damage caused by the broken gel fluid to the reservoir matrix. Natural sandstone cores were selected for the experiment, which was conducted at a reservoir temperature of 90 °C. First, the core was vacuumed and saturated with standard brine having a salinity of 85,000 mg/L (including divalent calcium and magnesium ions). The standard brine was forwardly displaced at the experimental temperature to determine the initial liquid permeability (*k*_1_). Subsequently, the broken gel filtrate was reversely injected to simulate the leak-off and invasion of the fluid into the formation during fracturing operations. Finally, the standard brine was forwardly displaced again until the pressure difference stabilized, and the damaged permeability (*k*_2_) was measured. Based on this, the final damage rate of the matrix permeability was calculated as shown in Equation (1) [35].

(1) $$D=\frac{k_{1}-k_{2}}{k_{1}}\times 100\%$$

where

$k_{1}$ is the initial liquid permeability of the core before contamination (10^−3^ μm^2^);

$k_{2}$ is the liquid permeability of the core after being contaminated by the fracturing fluid filtrate (10^−3^ μm^2^).

## Figures and Tables

**Table 1:** Formulations of different boron crosslinking agents.

No.	A/%	B/%	C/%	D/%	E/%	F/%
1	17	11	11	12	7	42
2	16.7	12.7	14.7	11.8	4.9	39.2
3	17	11	13	13	5	41
4	17	11	12	12	7	41
5	14.8	9.6	9.6	10.4	4.3	51.3
6	14.8	9.6	9.6	10.4	4.3	51.3
7	17	11	11	12	5	44
8	17	18	12	12	5	36
9	14.5	15.3	11	10.2	6.0	41.8
10	17	11	11	12	0	49
11	14.4	15.3	12.7	10.1	5.9	41.5

Note: A, B, C, D, E, and F represent the mass fractions of the respective components in the crosslinking agent system, with A through F corresponding to water, boric acid, sorbitol, triethanolamine, glyoxal, and sodium hydroxide, respectively.

**Table 2:** Crosslinking performance of different boron crosslinking agents.

No.	Crosslinking Time (s)	Gelation Time (s)	Hanging Ability
1	22.02	59.13	No
2	20.99	63.49	No
3	21.11	68.53	No
4	25.29	48.37	No
5	13.13	17.66	Yes
6	15.67	26.78	Yes
7	20.17	34.06	Yes
8	34.15	55.01	Yes
9	19.27	30.41	No
10	20.78	43.82	Yes
11	18.71	27.56	Yes

Note: The crosslinking and gelation times were recorded during the reaction with the 0.3% HPG solution. “Hanging Ability” indicates whether the crosslinked gel exhibits sufficient viscoelasticity to be hung (Yes) or not (No).

**Table 3:** Effect of HPG base fluid pH on crosslinking performance.

No.	pH	Crosslinking Time (s)	Gelation Time (s)	Hanging Ability
1	4	5.37	/	No gelation
2	4~5	6.07	12.42	Poor
3	7	18.71	27.55	Good
4	10	37.31	51.66	Good
5	11	38.14	58.96	Good, but gel degrades after 30 min
6	12	41.01	58.96	Good, but gel degrades after 30 min
7	12~13	43.99	64.24	Poor

Note: The tests were conducted using a 0.3 wt% HPG base fluid. “No gelation” indicates the system failed to form a three-dimensional network. “Degrades” refers to the loss of viscoelasticity and structural integrity after standing.

**Table 4:** Determination of the crosslinking time of 0.3% HPG solution with organoboron.

No.	Crosslinking Ratio	Crosslinking Time (s)	Gelation Time (s)	Hanging Ability
1	100:0.2	14.70	18.68	Brittle gel, partial hanging
2	100:0.3	12.39	14.83	Good hanging capability
3	100:0.4	10.74	12.93	Good hanging capability
4	100:0.5	10.03	12.05	Over-crosslinked, no hanging capability
5	100:0.6	10.12	12.56	Over-crosslinked, no hanging capability

**Table 5:** Determination of the crosslinking time of 0.4% HPG solution with organoboron.

No.	Crosslinking Ratio	Crosslinking Time (s)	Gelation Time (s)	Hanging Ability
1	100:0.2	8.51	22.49	Brittle gel, partial hanging
2	100:0.3	9.46	19.70	Good hanging capability
3	100:0.4	7.04	16.50	Good hanging capability
4	100:0.5	8.06	16.35	Good hanging capability
5	100:0.6	7.27	15.53	Over-crosslinked, no hanging capability

**Table 6:** Determination of the crosslinking time of 0.5% HPG solution with organoboron.

No.	Crosslinking Ratio	Crosslinking Time (s)	Gelation Time (s)	Hanging Ability
1	100:0.2	5.06	23.25	Brittle gel, partial hanging
2	100:0.3	4.88	24.12	Brittle gel, partial hanging
3	100:0.4	5.70	14.28	Good hanging capability
4	100:0.5	4.60	10.53	Good hanging capability
5	100:0.6	4.03	10.27	Over-crosslinked, no hanging capability

**Table 7:** Breaking of crosslinked hydroxypropyl guar (HPG) gel by ammonium persulfate (APS) at 90 °C.

No.	0.3%HPG/mL	Crosslinker Ratio	APS Concentration (%)	Apparent Viscosity (mPa·s)		
				0.5 h	1 h	2 h
1	100	100:0.3	0.001	unbroken gel	5.784	4.760
2			0.002	unbroken gel	3.218	
3			0.005	unbroken gel	1.554	
4			0.01	1.380		
5			0.02	1.330		
6			0.04	1.052		
7			0.08	1.03		

**Table 8:** Surface tension measurements of the gel-breaking fluids.

No.	Volume of 0.3% HPG (mL)	Crosslinker Concentration (%)	Breaker Concentration (%)	Flowback Additive Concentration (%)	Surface Tension (mN/m)
1	100	0.3	0.002	0	33.727
2					33.667
3					33.725
4	100	0.3	0.002	0.5	16.295
5					16.289
6					16.293

Notes: All surface tension measurements were performed at 25 °C using the ring method (Du Noüy ring tensiometer, if applicable). The surface tension of distilled water under identical conditions was 72.967 mN/m (reference value).

**Table 9:** Core parameters and test results.

Parameter	Value
Horizon	M2-2
Well interval (m)	1781.43–1781.46
Lithology description	Light gray fluorescent fine sandstone
Flowing medium	Standard brine
Experimental temperature (°C)	90
Core length (cm)	5.432
Core diameter (cm)	2.411
Pore volume (cm^3^)	2.116
Porosity (%)	8.534
Gas permeability (×10^−3^ μm^2^)	0.39500
Initial permeability k_1_ (×10^−3^ μm^2^)	0.09125
Post-damage permeability k_2_ (×10^−3^ μm^2^)	0.07231
Matrix permeability damage rate (%)	20.76

Notes: Standard brine composition: 2.0 wt% KCl + 5.5 wt% NaCl + 0.45 wt% MgCl_2_ + 0.55 wt% CaCl_2_ in deionized water.

**Table 10:** Determination of residue content.

No.	Volume of 0.3% HPG (mL)	Crosslinker (mL)	Breaker Concentration (%)	Residue Content (mg/L)
1	100	0.3	0.03	290
2	100	0.3	0.03	280
3	100	0.3	0.03	278

Notes: Breaker: ammonium persulfate (APS). Gel breaking was performed at 90 °C. Values are from three parallel measurements under identical conditions.

## Data Availability

The original contributions presented in the study are included in the article further inquiries can be directed to the corresponding author.

## Abbreviations

The following abbreviations are used in this manuscript:

TEOA	Triethanolamine
HPG	Hydroxypropyl guar gum
